# WGCNA-Based DNA Methylation Profiling Analysis on Allopurinol-Induced Severe Cutaneous Adverse Reactions: A DNA Methylation Signature for Predisposing Drug Hypersensitivity

**DOI:** 10.3390/jpm12040525

**Published:** 2022-03-24

**Authors:** Lin Cheng, Bao Sun, Yan Xiong, Lei Hu, Lichen Gao, Ji Li, Hongfu Xie, Xiaoping Chen, Wei Zhang, Hong-Hao Zhou

**Affiliations:** 1 State Key Laboratory of Ophthalmology, Zhongshan Ophthalmic Center, Sun Yat-sen University, Guangzhou 510060, China; 2 School of Ophthalmology and Optometry, Eye Hospital, Wenzhou Medical University, Wenzhou 325027, China; 3 Department of Clinical Pharmacology, Xiangya Hospital, Central South University, Changsha 410008, China; scy_csu2016@csu.edu.cn (B.S.); yanzi2546@foxmail.com (Y.X.); hulei@phpku.edu.cn (L.H.); chenxiaoping@csu.edu.cn (X.C.); csuzhangwei@csu.edu.cn (W.Z.); 4 Hunan Key Laboratory of Pharmacogenetics, Institute of Clinical Pharmacology, Central South University, Changsha 410078, China; 5 Department of Pharmacy, Department of Oncology, Cancer Institute, Changsha Central Hospital, Changsha 410004, China; 2018050699@mail.usc.edu.cn; 6 Department of Dermatology, Xiangya Hospital, Central South University, Changsha 410008, China; liji_xy@csu.edu.cn (J.L.); xiehongfu@csu.edu.cn (H.X.)

**Keywords:** drug hypersensitivity, epigenetics, DNA methylation, allopurinol-induced severe cutaneous adverse reactions (SCARs), weighted gene co-expression network analysis (WGCNA), ATG13, microRNAs

## Abstract

Background: The role of aberrant DNA methylation in allopurinol-induced severe cutaneous adverse reactions (SCARs) is incompletely understood. To fill the gap, we analyze the DNA methylation profiling in allopurinol-induced Stevens-Johnson syndrome (SJS) and toxic epidermal necrolysis (TEN) patients and identify the DNA methylation signature for predisposing allopurinol hypersensitivity. Methods: Genome-scale methylation analysis was conducted using the Illumina^®^ HumanMethylation450 BeadChip. Weighted Gene Co-Expression Network Analysis (WGCNA) was utilized to analyze the data. Results: A total of 21,497 annotated promoter regions were analyzed. Ten modules were identified between allopurinol hypersensitivity and tolerance, with turquoise and yellow modules being the most significant correlation. ATG13, EPM2AIP1, and SRSF11 were the top three hub genes in the turquoise module. MIR412, MIR369, and MIR409 were the top three hub genes in the yellow module. Gene Ontology (GO) analysis revealed that the turquoise module was related to the metabolic process in intracellular organelles and the binding of various compounds, proteins, or nucleotides. The yellow module, however, was related to stimulus sensory perception in cytoskeletal elements and the activity of the receptor or transducer. Conclusion: DNA methylation plays a vital role in allopurinol-induced SCARs. DNA methylation profiling of SJS/TEN is significantly related to autophagy and microRNAs (miRNAs).

## 1. Introduction

Previously we found that allopurinol-induced severe cutaneous adverse reactions (SCARs) are strongly associated with HLA-B*58:01, with 94.57% of patients carrying the allele [1]. HLA-B*58:01 is the biomarker for allopurinol-induced SCARs. However, understanding how allopurinol triggers the disease and brings forth autoimmune reactions is not complete. The current concept is the HLA-dependent cytotoxic T cell-mediated delayed-type IV hypersensitivity reactions. The drug covalently binds to the major histocompatibility complex (MHC) in the antigen-presenting cells and stimulates the specific cytotoxic T lymphocytes and cascade release of cytokines, such as the perforin, granzyme, and granulysin [2,3]. These cytokines or chemokines act upon keratinocytes and epithelia to trigger the cytotoxic autoimmune reactions resulting in massive death of keratinocytes and mucous cells. In Stevens-Johnson syndrome (SJS) and toxic epidermal necrolysis (TEN), T lymphocytes behave autologously cytotoxic to the homologous target cells.

We found that not all patients carrying HLA-B*58:01 develop SCARs, nor do SCARs patients occur exclusively in HLA-B*58:01 carriers. Why do some carriers have immune privilege? We already know that drug hypersensitivity is a multi-gene interaction complex disease and that many genes and molecules are involved [4]. The mechanism of drug hypersensitivity is not fully elaborated and demands promising targets for treating drug-induced SCARs. As such, growing evidence supports that some epigenetic regulators are risk factors for some autoimmune diseases. For instance, histone modification and DNA methylation are risk factors for systemic lupus erythematosus (SLE), rheumatoid arthritis (RA), multiple sclerosis (MS), and other diseases [5,6,7,8,9]. Recent studies have shown that drug hypersensitivity was closely related to microRNAs (miRNAs) [10,11]. Another investigation described the overexpression of miRNA-18a-5p inhibited the expression of B-cell lymphoma/leukemia-2-like protein 10 (BCL2L10), which is an endogenous protein that inhibits apoptosis. The overexpression of miRNA-18a-5p led to endogenous keratinocyte apoptosis in TEN. Plasma levels of miRNA-18a-5p could be a biomarker for TEN [12]. These data show that epigenetic modifications are involved in autoimmune diseases and drug hypersensitivity.

DNA methylation is an essential part of the epigenetic landscape that may play a critical role in drug hypersensitivity. DNA methylation refers to the CpG dinucleotide 5′ cytosine transformation into 5′ methylcytosine under the catalysis of DNA methyltransferase (DNMTs). This modification does not change DNA gene sequences but regulates gene expression. CpG islands are stretches of DNA 500–1500 bp long with a CG:GC ratio of more than 0.6, GC content greater than 50% [13], and located near the CAT box (CCAAT) to regulate transcription efficiency. CpG islands contain abundant cytosine (C) and guanine (G) connected with a phosphate bond (*p*), and they often are not methylated [14,15]. In mammals, CpG sequences are rare in the genome because the cytosines in such an arrangement tend to be methylated. They often appear in specific genes, especially the promoter region, the first exon region, and the end of the housekeeping genes [13]. A total of 50–70% of human gene promoters have CpG islands [16].

There are approximately 40,000 CpG islands in mammalian genomes. In the healthy human genome, the CpG sites of CpG islands are usually in a non-methylation status, while the CpG sites outside the CpG islands are usually methylated. This methylation state can form stable retention during cell division. The table will be turned over in tumorigenesis. In tumor suppressor genes, the CpG islands located in the promoter regions are hypermethylated, leading to the compressed structure of chromatin and inactivation of tumor suppressor genes [17]. Moreover, CpG island methylation is also involved in embryonic development, transcription, chromatin structure, X-chromosome inactivation, genomic imprinting, chromosome stability, and other diseases’ biological processes [18,19].

To better elucidate the DNA methylation signature for predisposing drug hypersensitivity, we aim to examine the whole-genome DNA methylation profiling of allopurinol-induced SCARs and allopurinol-tolerant patients. We use Weighted Gene Co-Expression Network Analysis (WGCNA) and Gene Ontology (GO) annotation to analyze the methylation data in allopurinol-induced SCARs and find the new pathways that might contribute to the disease. The differentially methylated genes might reveal the potential pathways associated with allopurinol hypersensitivity and offer promising avenues of exploration for the future development of therapeutics.

## 2. Materials and Methods

### 2.1. Patients

The inclusion criteria were as follows. All participants had to be of Han Chinese descent. The allopurinol-induced SCARs patients, including SJS, TEN, and SJS/TEN overlap, were diagnosed by dermatologists according to Roujeau criteria [20]. SCARs had to occur within three months of allopurinol use, with diminished or relieved symptoms upon withdrawal. SJS and TEN are severe reactions and are commonly overlap in the clinic. SJS and TEN are characterized by fever and mucocutaneous lesions (mouth, lips, genital, and anal regions), which lead to epidermal death and sloughing. SJS is defined as skin detachment < 10% of the total body surface area, SJS/TEN overlap as 10–30%, and TEN as >30% skin detachment. The control patients were allopurinol-tolerant, defined as patients who took allopurinol for a least three months but showed no evidence of cutaneous adverse reaction. Patients were enrolled from 20 different hospitals across China.

The exclusion criteria were patients with a medical history of bone marrow transplantation, chemotherapy, or cancer. Patients who met the inclusion criteria but did not comply with the exclusion criteria were enrolled.

Our study enrolled 15 allopurinol-induced SCARs patients (3 TEN, 2 SJS/TEN overlap, and 10 SJS) and 20 allopurinol-tolerant patients. The characteristics of included patients are shown in a previous publication [21].

### 2.2. Blood Sample Collection, Genomic DNA Extraction, and Methylation Beadchip Assay

Two ml peripheral blood samples were collected from the patients to extract the genomic DNA. The DNA quality was checked in 0.8% agarose gel electrophoresis, which should have clear bands, and the length is longer than 10 kb without noticeable degradation. The OD260/280 value was between 1.7–2.1; the DNA concentration was no lower than 50 ng/uL; the total DNA was no less than 2 ug.

The Illumina^®^ HumanMethylation450 BeadChip assay (35 chips) was conducted following the manufacturer’s manual. The principle was to use sulfite to treat genomic DNA. If the C is methylated, it will remain unchanged; if C is not methylated, it will be bisulfite conversed to U, and be further converted to T by PCR amplification. Briefly, the genomic DNAs were bisulfite conversed and denatured and neutralized. Double strand DNAs were broken into fragmentation, precipitated, and hybridized with microarray. The hybridized microarray was washed, the single bases were extended and stained. Finally, the microarray was scanned, and the data were extracted. The methylation array has 484,660 microarray probes, covering 99% of human RefSeq genes, with an average of 17 CpG sites per gene region distributed across the promoter, 5′UTR, first exon, gene body, and 3′UTR of each gene. It covers 96% of CpG islands, island shores, and those regions flanking island shores (island shelves) [22].

### 2.3. Analysis Software

The WGCNA package and the necessary libraries in the Bioconductor of the R software were used to analyze the regulatory network of methylation profiling. The differentially methylated genes were identified. The functional annotations and analysis were conducted using GO databases (http://geneontology.org/, accessed on 25 October 2021) for three aspects: Molecular Function, Cellular Component, and Biological Process.

### 2.4. Screening Differentially Methylated Positions and Regions

Analyzing differentially methylated positions (DMPs) can help find the significantly differentially methylated CpG single position. To find the biological significance of these significantly methylated CpG positions, we analyzed it with the genome position, which was differentially methylated regions (DMRs). DMRs are divided into genomic tiling, gene, promoter, and CpG island [23]. Screen DMRs can help to explain how the significant methylation modifications cause gene expression regulation, such as the hypermethylation in promoter regions results in gene suppression [18].

The methods that analyze the DMRs include Illumina Methylation Analyzer (IMA) [24], City of Hope CpG Island Analysis Pipeline (COHCAP) [25], The Chip Analysis Methylation Pipeline (ChAMP) [26], and RnBeads [27]. Our analysis combines the linear fitting algorithm limma [28], COHCAP, and RnBeads, and uses a Combined Rank to sort DMPs and DMRs. The Combined Rank uses a comprehensive sorting algorithm with methylation 𝛽 value, paired T-test (*p*-value), and False Discovery Rate (FDR). The higher the sort, the higher the reliability of DMPs and DMRs, and vice versa.

### 2.5. Algorithm of Weighted Gene Co-Expression Network Analysis (WGCNA)

Each co-expression network corresponds to an adjacency matrix. The adjacency matrix represents the strength of the connection between each pair of genes. The chip can only detect the methylation of a single CpG site, while the β value and M value can to measure the degree of methylation [29]. Therefore, we designed a variety of methods to construct and screen methylation co-expression networks, including using a single DMP or DMR, using β or M values to measure methylation, and using methylation data adjusted network topology parameters, etc. Finally, we constructed a co-model of the “unsigned” type (see Formula (1)) by using the M values in the promoter regions and the β value of 14. The soft threshold was set to 14 to achieve a maximum balance between free topology fitting and connectivity.

(1) $$\begin{array}{c} a_{ij}=\left|cor{\left(x_{i},x_{j}\right)}^{\beta^{\prime }}\right| \end{array}$$

($x_{i}$ and $x_{j}$ are the degree of methylation in the upstream promoter region of i and j genes. cor: correlation; $\beta^{\prime }$ is the soft-thresholding power for constructing a co-expression network. $\beta^{\prime }$ sets the gene network to obey a scale-free distribution is a correlation index, which is different from the β value that measures the degree of methylation).

### 2.6. The Definition of Dissimilarity Measurement in WGCNA

WGCNA uses dissimilarity to cluster, namely, measure the dissimilarity between different genes. The “topological overlap” between two genes reflects the strength of the connection between them. The definition of Topological Overlap Matrix (*TOM*) is defined below [30,31,32]:

(2) $$\begin{array}{c} TOM_{ij}=\frac{\sum_{u\neq i,j}a_{iu}a_{uj}+a_{ij}}{\min\left(k_{i},\;k_{j}\right)+1\;-\;a_{ij}} \end{array}$$

To convert this matrix into dissimilarity to measure gene distance, TOM was converted into dissimilarity *TOM* (*distTOM*) by $\;\begin{array}{c} distTOM_{ij}=1-TOM_{ij} \end{array}$. u was a gene other than i and  j. The dissimilarity values calculated by TOM can produce specific modules. We analyzed the highly connected genes (hub genes) in the specific modules together with the biological annotations and aimed to uncover the genes or signal pathways with clinical significance.

## 3. Results

### 3.1. The Methylation Array Data Were Quality Controlled and Normalized

The raw data of the 35 methylation chips were quality-checked. Quality-check suggested the data were of high quality and could be normalized. The “Function Normalization” algorithm was used to normalize the IDAT raw data files in the methylation array according to the probe categories, methylated and unmethylated signals, and gender [33]. After normalization, the β and M values were more evenly distributed, the chip background noise and the error between the chipset was minimized, and the reliability of the subsequent DMPs screening was improved.

(3) $$\begin{array}{c} \beta =\frac{M}{M+U+\alpha } \end{array}$$

(4) $$\begin{array}{c} M=\log2\left(\frac{M+\alpha }{U+\alpha }\right) \end{array}$$

(5) $$\begin{array}{c} M=\log2\left(\frac{\beta }{1-\beta }\right) \end{array}$$

β and M values were utilized to measure the methylation of each CpG site. The β value is the methylation percentage with a range [0, 1]. The closer it is to 1, the higher degree of methylation, and vice versa. In Formula (3), M and U values represent methylated and unmethylated signal intensity; α is an arbitrary predetermined threshold set at 100. The M value (not the M value in Formula (3)) was initially defined in Formula (4) [29], and α in the formula was defined as 1.

Due to more than 95% of the probes in the chip having signal strengths higher than 1000, the effect of the α values in Formulas (3) and (4) are negligible. Therefore, the M value is regarded as the logit conversion of the β value [34], see Formula (5). After logit conversion, the β value with the value range of [0, 1] can be converted into the M value with [−∞,+∞] range. The β value describes the percentage of methylation, which is relatively robust but rough to describe the degree of methylation due to close value of positions or regions. Therefore, β value is converted to M value with a broad range. After conversion, the M value has a high sensitivity to methylation changes. Therefore, our study uses both the β value and the M value to measure changes in the degree of methylation. The closer the M value to −∞, the lower methylation of the CpG position. The closer the M value to +∞, the higher methylation of the CpG position.

### 3.2. Screening Differentially Methylated Positions and Regions

**Figure 1:**
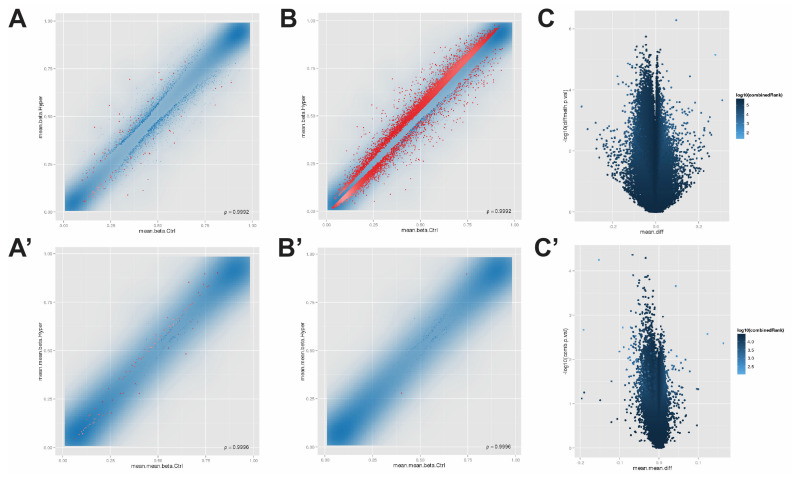
The detected DMPs or DMRs of control vs. hypersensitivity reactions. (**A**) Top 100 DMPs. The *x-* and *y*-axis are the mean β values of the control group against the hypersensitivity reactions group. The red points represent the top 100 DMPs. (**B**) DMPs with a rank higher than the threshold. The *x-* and *y*-axis have the same meaning as (**A**). The red points represent DMPs higher than the threshold. (**C**) The volcano plot of whole-genome CpG sites. The *x-* and *y*-axis denotes the mean differential β values and negative logarithm transformed *p*-value, respectively. The asymmetrical distribution of (**C**) revealed that the detected hypermethylated and hypomethylated sites were in different regions, which indicates the high quality of the methylation array. (**A’**) Top 100 DMRs in promoter regions. The *x-* and *y*-axis are the mean β values of the control group against the hypersensitivity reactions. The red points represent the top 100 DMRs. (**B’**) Promotor region DMRs with a rank higher than the threshold. The *x-* and y-axis have the same meaning as (**A’**). The red points represent DMRs higher than the threshold. (**C’**) The volcano plot of whole-genome promoter regions. The *x-* and *y*-axis denotes the mean differential β values and negative logarithm transformed *p*-value, respectively. The asymmetrical distribution of Figure (**C’**) revealed that the detected hypermethylated and hypomethylated sites were in different regions, which indicates the high quality of the methylation array.

After data quality control and normalization, 465,686 CpG sites were obtained and were further analyzed to identify the DMPs and DMRs. The 465,686 CpG sites with the top 100 DMPs in “combinedRank” in red were shown in Figure 1A. DMPs with ranks higher than the threshold (CpG site 137,828; Tiling 9483; promoter 159; genes 3334; CpG island 494) in red were shown in Figure 1B. The whole-genome CpG sites were volcano plotted with differential β values and negative logarithm transformed *p*-values (Figure 1C).

Our study focuses on the DMRs in the promoter region since it is closely related to the regulation of gene transcription [18]. Among 465,686 CpG positions, 30,911 are in the promoter region. After removing the promoters that are not translated into proteins, including ribosomal RNA promoters, pseudogene promoters, and unannotated promoters, we finally analyzed 21,497 annotated promoter regions (Table S1). The DMRs in the promoter region detected from hypersensitivity patients against allopurinol control patients (20 vs. 15 patients) were shown in Figure 1A’, with the top 100 DMRs in “combinedRank” shown in red. The DMRs with ranks higher than the threshold were shown in red (Figure 1B’). The whole-genome promoter regions were volcano plotted with the mean differential β values and negative logarithm transformed *p*-values (Figure 1C’).

### 3.3. Selection of Parameters for WGCNA

WGCNA is used to find functionally similar genes or connected gene loci. We set the cut-off value as 0.15 in WGCNA analysis and obtained 10 different modules. Combining “Gene Significance, *GS*” (see Formula (6)) and the number of genes in each module, we got the most significant modules of turquoise and yellow.

(6) $$\begin{array}{c} GS_{i}=-log2\left(p-value_{i}\right) \end{array}$$

(In this formula, p−value is the difference in the methylation level of the promoter region between the “normal group” and the “hypersensitivity group” obtained according to the linear fitting algorithm *limma* [28]. The greater the GS, the greater the significance, and vice versa).

**Figure 2:**
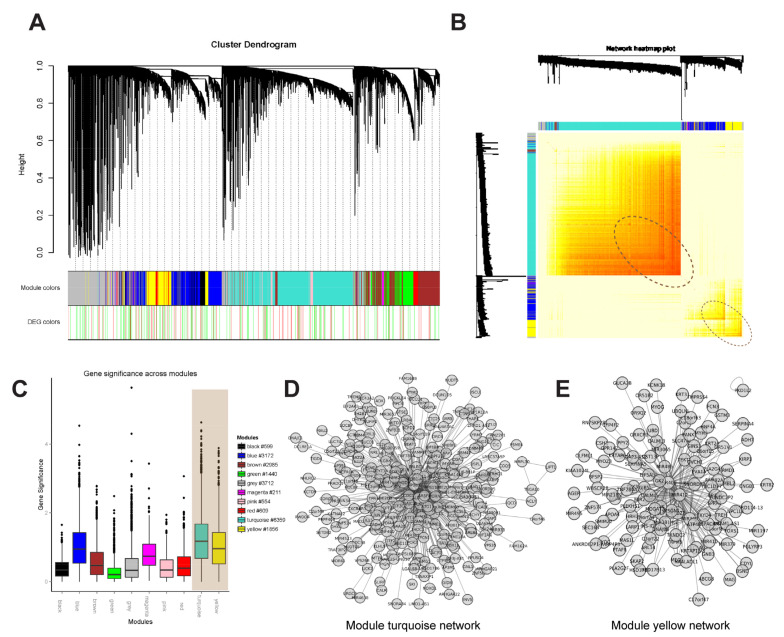
Weighted correlation network analysis (WGCNA) to identify gene modules highly correlated with methylation traits of allopurinol-induced severe cutaneous adverse reactions. (**A**) Cluster dendrogram and modules of whole-genome methylation correlation network. The top part is the cluster dendrogram of selected 21,497 promoters. The first color frame denotes the distribution of different modules, and the second color frame shows the significant methylated promoters. The hypermethylated and hypomethylated promoters are marked with red and green in the hypersensitivity reactions group, respectively. The threshold is defined as *p*-value < 0.02. (**B**) The two-dimension cluster dendrogram of top 1000 promoter regions. The top 1000 promoters are significant with the *p*-values smaller than 0.01. The left and top bars show the cluster dendrograms and modules of the top 1000 promoters with the same colors in Figure 2A. The middle heatmap represents the correlation of paired genes. The color close to red and the color close to white denotes the high and low correlation, respectively. The turquoise and yellow modules have a high self-concentration and contain a large number of significantly methylated promoters. (**C**) Histogram of module GS. The *x-*axis and *y*-axis denotes modules and GS values. The legend shows the numbers of promoters in each module. GS: Gene Significance. (**D**) The correlation network map of the promoter region in the turquoise module. The connection correlation was set to 0.22, and the correlation coefficient was >0.89. (**E)** The correlation network map of the promoter region in the yellow module. The connection correlation was set to 0.20, and the correlation coefficient was >0.89.

To better illustrate the turquoise and yellow modules, we made cluster dendrograms with modules of whole-genome methylation correlation networks (Figure 2A,B) and a histogram with module Gene Significance (GS) (Figure 2C). Turquoise and yellow modules had the most significant correlation with allopurinol hypersensitivity (Figure 2B, circled highlighting the dark yellow color) and high Gene Significance values (Figure 2C, highlighted in grey). In these two modules, we defined the link relevance as 0.22 and 0.20 in the turquoise and yellow, respectively, to demonstrate the association network (both correlation indexes > 0.89). The full lists of promoter genes in the turquoise and yellow modules were shown in Tables S2 and S3, respectively.

### 3.4. Hub Genes Selection

Hub genes indicate genes with more connected nodes than other genes in a network. In methylation co-expression networks, hub genes are usually at the core of the network, reflecting their key regulatory role in biological processes or pathways. Therefore, a network composed of several hub genes can represent the characteristics of a network, such as specific signal transduction pathways and metabolic pathways.

**Figure 3:**
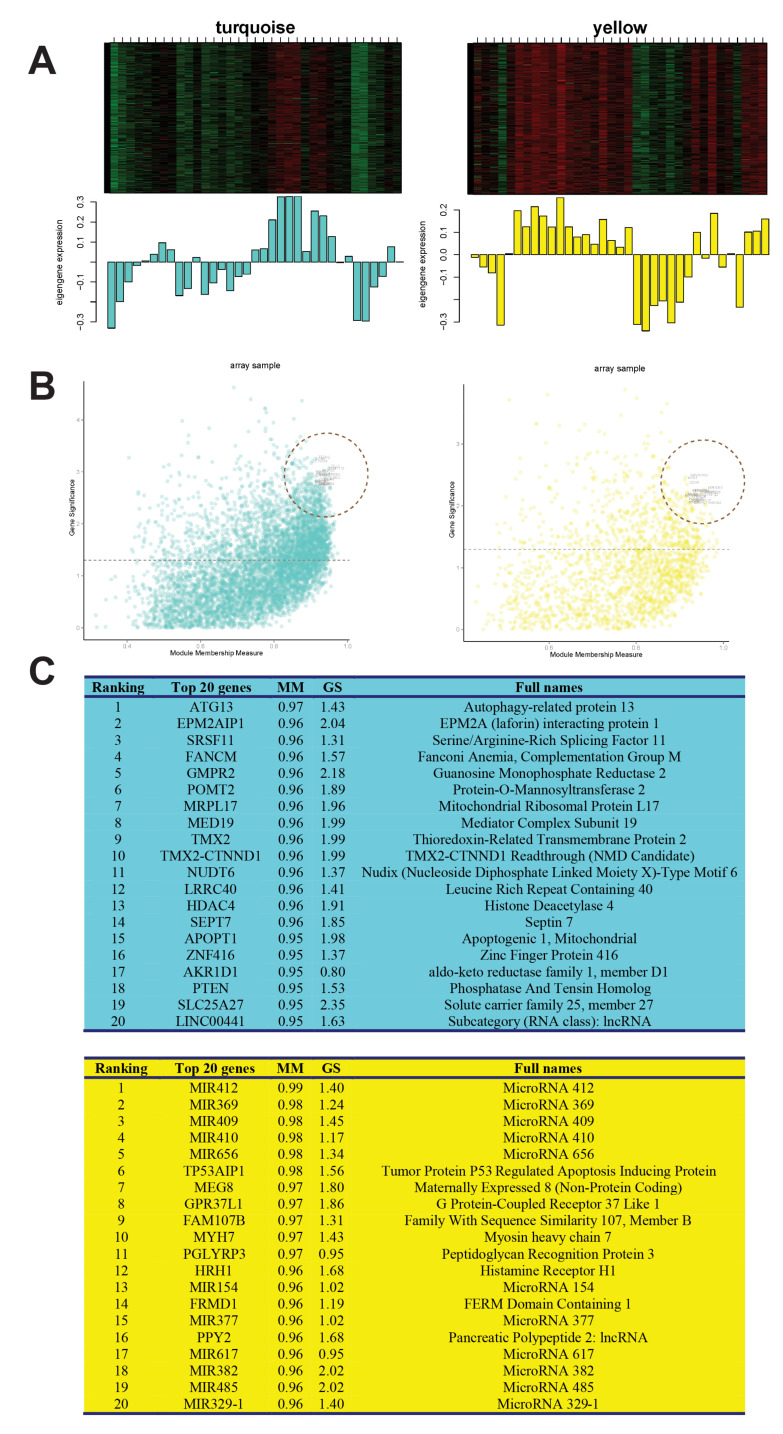
The hub genes in the turquoise and yellow modules. (**A**) The eigengene plots of turquoise and yellow modules. The top denotes the heatmaps of 35 samples, each row represents a promoter region, and each column represents a sample. The bottom denotes the bar plots of eigengene in each sample. The turquoise color represents the turquoise module, and the yellow color represents the yellow module. (**B**) The scatter plots of hub genes in turquoise and yellow modules. The *x*-axis represents the inner-modular connectivity, and the *y*-axis represents the gene significance. The dashed line points out the threshold (*p*-value = 0.05). The top 20 hub genes with the highest inner-modular connectivity are marked. (**C**) The top 20 hub genes in the turquoise module and yellow module. The background color represents the different modules. MM: Membership Measurement; GS: Gene Significance.

Using the “intramodular connectivity” and “gene significant parameters” in the turquoise and yellow modules, we displayed the module eigengenes and hub genes (Figure 3A,B). Some promoter regions were hypermethylated, while others were hypomethylated in turquoise and yellow modules (Figure 3A). The scatter plots of hub genes in the turquoise and yellow modules show how these genes were distributed (Figure 3B). The top 20 hub genes with high connectivity and high significance in the turquoise and yellow modules were marked in the corresponding positions in the dashed circles, from high to low in two modules (Figure 3C). The greater the degree of connectivity (Membership Measurement, MM), the greater the number of genes connected to it, namely, more genes are connected to hub genes. Gene Significance (GS) indicates the degree of significant difference in genes; the larger the GS, the greater the significant difference. GS and MM are close to a positive correlation.

In Figure 3B, the *y*-axis is GS and the *x*-axis is *MM*. The calculation formula of *MM* is as follows:

(7) $$\begin{array}{c} MM_{yellow}\left(i\right)=cor\left(x_{i},ME_{yellow}\right) \end{array}$$

**Figure 4:**
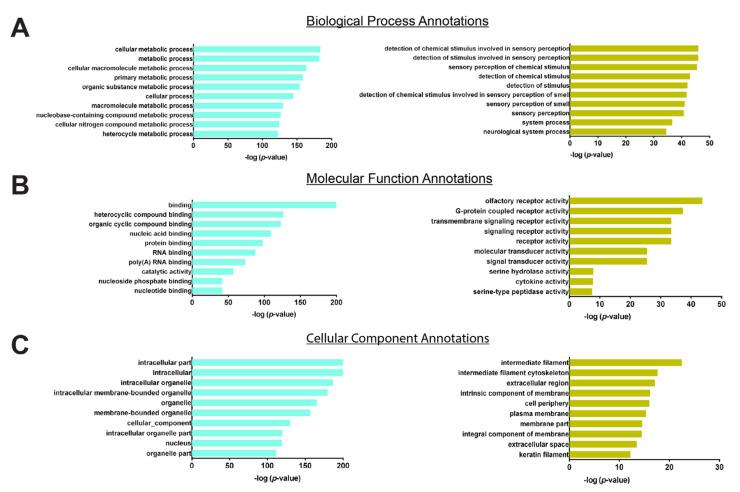
The top 10 gene ontology annotations in turquoise and yellow modules. The turquoise color denotes the turquoise module; the yellow color denotes the yellow module. (**A**) The top 10 Biological Process Annotations in turquoise and yellow modules. (**B**) The top 10 Molecular Function Annotations in turquoise and yellow modules. (**C**) The top 10 Cellular Component Annotations in turquoise and yellow modules.

To analyze the biological functions corresponding to the turquoise and yellow modules, “Gene Ontology GO annotations were made for these two modules, including “Biological Process (BP)”, “Molecular Function (MF)”, and “Cellular Component (CC)” (Figure 4). The *p*-value refers to the significance of the gene set annotated in the yellow and turquoise modules. GO analysis revealed that the turquoise module was related to the metabolic process in intracellular organelles and the binding of various compounds, proteins, or nucleotides. The yellow module, however, was related to stimulus sensory perception in cytoskeletal elements and the activity of the receptor or transducer.

## 4. Discussion

This is the first study using WGCNA method to study allopurinol hypersensitivity to the best of our knowledge. We established the co-expression network of the DMRs in the promoter region. The characteristic of the network is excellent, indicating the high reliability of the data. The DMPs and DMRs suggest gene methylation is another crucial factor associated with allopurinol-induced SJS/TEN.

WGCNA is primarily used to build a co-expression network based on the expression levels of transcriptome [30,31,32]. Horvath et al. first proposed a gene co-expression network for DNA methylation in the aged human brain and blood tissue [35]. WGCNA is a widely used data mining method for genomic analysis. Instead of relating individual genes to phenotype, WGCNA focuses on the relationship between a few modules and the trait, which significantly alleviates the multiple testing problem inherent in microarray data analysis [36]. The WGCNA algorithm assumes that the gene network obeys scale-free topology and defines a gene co-expression correlation matrix and the adjacent gene network function. Then, the dissimilarity coefficient in different nodes is calculated to build a hierarchical clustering tree. The different branches of the clustering tree represent different gene modules, and those genes with a similar degree of similarity are clustered into one module. The genes in the same module have a high co-expression similarity, while those in the different modules have a relatively low co-expression similarity. Finally, the relationship between the modules and phenotypes or disease are explored, and the therapeutic targets for disease or genetic networks are identified.

We found that the modules of turquoise and yellow have a high correlation with allopurinol hypersensitivity. ATG13 (autophagy-related protein 13), EPM2AIP1 (EPM2A (laforin) interacting protein 1), and SRSF11 (serine and arginine-rich splicing factor 11) are the top three significant genes in the turquoise module (Figure 3C). ATG13 is an important autophagy gene that works together with ULK1 (Unc-51-like autophagy activating kinase 1) to regulate the TOR kinase signaling pathway in autophagy and moderate the ATG13-ULK1-RB1CC1 (RB1 inducible coiled-coil 1) complex. A ULK1/2 binding-deficient ATG13 variant resulted in diminished but not completely abolished autophagic activity in HEK 293 cells, highlighting a peptide motif at the C terminus of ATG13 is required for the binding of ULK1/2 to initiate the autophagy [37]. Atg13 knockout resulted in a fetal loss in mice, and those embryos showed growth retardation and myocardial growth defects. Atg13 deficiency blocked autophagosome formation at an upstream step in fibroblasts and showed enhanced TNF-α-induced apoptosis. This phenomenon was not seen in other Atg-deficient mice nor by simultaneous deletion of Ukl1 and Ulk2 [38]. ATG13 is most likely an important gene that mediates SJS/TEN autophagy and immune recognition. The EPM2AIP1 gene is a vital gene regulating laforin protein, and the laforin protein transmits chemical signals and breaks down unneeded or abnormal proteins. EPM2AIP1 plays a crucial role in Lafora disease (LD), a fatal form of progressive myoclonus epilepsy characterized by neurodegeneration and the presence of intracellular polyglucosan inclusions (Lafora bodies) in different tissues [39]. Moreover, EPM2AIP1 is glycogen synthesis-associated. The absence of Epm2aip1 in mice impaired allosteric activation of glycogen synthesis, decreased hepatic glycogen synthesis, increased liver fat, caused hepatic insulin resistance, and protected against age-related obesity. On the contrary, another study showed Epm2a knockout mice had enhanced insulin response [39,40]. Despite disparity, it suggests that EPM2AIP1 is a regulator of insulin sensitivity. SRSF11 ranks third place in the turquoise module. SRSF11 plays a role in pre-mRNA processing and alternative splicing which confers a growth advantage [41,42]. SRSF11 loss leads to aging-associated cognitive decline [43]. Mouse cocaine intake induced H3K36me3 enrichment and alternative splicing of Srsf11, showing Srsf11 has the function to regulate downstream splice events to augment cocaine-reward behavior [44].

The top hub genes in the yellow module are miRNAs, such as MIR412, MIR369, MIR409, etc., which shows that miRNAs play a crucial role in SJS/TEN. In addition, TP53AIP1 (tumor protein p53 regulated apoptosis-inducing protein, ranks sixth in the yellow module) participates in p53-mediated cell apoptosis. p53, a tumor suppressor gene, is involved in regulating keratinocyte apoptosis. p53 mutations are seen in skin precancers and sun-exposed skin, which harbors thousands of p53-mutant keratinocyte clones [45]. Therefore we assume that the p53-mediated keratinocyte necrosis pathway may play a role in drug hypersensitivity.

Our study has limitations. We found that DNA methylation traits play an important role in regulating drug hypersensitivity. But we do not know the different methylation status leads to the expression of different genes, thus affecting drug metabolism and causing drug hypersensitivity, or if the different methylation status is the outcome of drug hypersensitivity. Moreover, the genes identified by WGCNA need to be further investigated to verify their function in allopurinol hypersensitivity. Ideally, the methylation data could be analyzed together with RNA-seq data to explore the molecular mechanism of drug hypersensitivity. RNA-seq data will be further addressed in the future.

## 5. Conclusions

The methylation profile in allopurinol-induced SCARs patients (TEN, TEN/SJS, and SJS) is significantly different from allopurinol-tolerant patients. This indicates that methylation plays an important role in drug hypersensitivity. WGCNA identifies the turquoise and yellow modules that are highly correlated with methylation traits of allopurinol-induced SCARs patients. ATG13 and MIR412 are the most significant hub genes in the turquoise and yellow modules. The autophagy signaling pathway mediated by ATG13 may be the critical pathway that mediates SJS/TEN autophagy and immune recognition in SJS/TEN. The methylation changes of miRNAs and pre-mRNA splicing factor genes suggest that the noncoding RNAs are also involved in the epigenetic regulation of allopurinol hypersensitivity. p53 may be involved in the regulation of keratinocyte apoptosis. GO analysis suggests allopurinol hypersensitivity differs from allopurinol tolerance in multiple biological pathways, such as stress response, binding, energy metabolism, and receptor activity. The small molecules or protein interventions that target autophagy and miRNAs/pre-mRNA splicing may provide promising targets for treating drug hypersensitivity. Our study provides new ideas for the prevention and treatment of allopurinol-induced SJS/TEN. More data are needed to test the effectiveness of these potential treatment targets in patients.

## Data Availability

The data presented in this study are available in supplementary material here.

## Supplementary Materials

The following supporting information can be downloaded at: https://www.mdpi.com/article/10.3390/jpm12040525/s1, Table S1: The 21,497 annotated promoter regions used for the analysis of differentially methylated regions (DMRs); Table S2: The full list of promoter genes in the turquoise module. Table S3: The full list of promoter genes in the yellow module.
